# Magneto-Rheological Fluid Assisted Abrasive Nanofinishing of β-Phase Ti-Nb-Ta-Zr Alloy: Parametric Appraisal and Corrosion Analysis

**DOI:** 10.3390/ma13225156

**Published:** 2020-11-16

**Authors:** Sunpreet Singh, Chander Prakash, Alokesh Pramanik, Animesh Basak, Rajasekhara Shabadi, Grzegorz Królczyk, Marta Bogdan-Chudy, Atul Babbar

**Affiliations:** 1 Department of Mechanical Engineering, National University of Singapore, Singapore 119077, Singapore; snprt.singh@gmail.com; 2 School of Mechanical Engineering, Lovely Professional University, Phagwara, Punjab 144411, India; 3 Department of Mechanical Engineering, Curtin University Australia, Perth 6102, Australia; alokesh.pramanik@curtin.edu.au; 4 Adelaide Microscopy, The University of Adelaide, Adelaide, SA 5005, Australia; animesh.basak@adelaide.edu.au; 5 Unité Matériaux et Transformations CNRS UMR 8207, Université de Lille, 59000 Lille, France; rajashekhara.shabadi@univ-lille.fr; 6 Department of Mechanical engineering, Opole University of Technology, 45-758 Opole, Poland; m.bogdan-chudy@po.edu.pl; 7 Mechanical Engineering Department, Shree Guru Gobind Singh Tricentenary University, Gurugram 122505, India; atulbabbar123@gmail.com

**Keywords:** β-phase TNTZ alloy, nano-finishing, magnetic abrasive finishing, surface roughness, material removal, optimization, parametric appraisal

## Abstract

The present work explores the potential of magneto-rheological fluid assisted abrasive finishing (MRF-AF) for obtaining precise surface topography of an in-house developed β-phase Ti-Nb-Ta-Zr (TNTZ) alloy for orthopedic applications. Investigations have been made to study the influence of the concentration of carbonyl iron particles (CIP), rotational speed (Nt), and working gap (Gp) in response to material removal (MR) and surface roughness (Ra) of the finished sample using a design of experimental technique. Further, the corrosion performance of the finished samples has also been analyzed through simulated body fluid (SBF) testing. It has been found that the selected input process parameters significantly influenced the observed MR and Ra values at 95% confidence level. Apart from this, it has been found that Gp and Nt exhibited the maximum contribution in the optimized values of the MR and Ra, respectively. Further, the corrosion analysis of the finished samples specified that the resistance against corrosion is a direct function of the surface finish. The morphological analysis of the corroded morphologies indicated that the rough sites of the implant surface have provided the nuclei for corrosion mechanics that ultimately resulted in the shredding of the appetite layer. Overall results highlighted that the MRF-AF is a potential technique for obtaining nano-scale finishing of the high-strength β-phase Ti-Nb-Ta-Zr alloy.

## 1. Introduction

Roughly 80% of biomedical implants are developed using metallic materials, including stainless steel, cobalt-chromium, Nitinol, and titanium alloys. This is mainly due to the fact that the metallic biomaterials play a remarkable role in the recovery of dysfunctional organs and improving the life of human beings [1]. Further, the need of the Ti-based biomaterials is consistently growing due to the rapid increase in the population of the elderly population, road accidents, and sports injuries. Ti-alloy based biomaterials have been used for the development of organs due to their excellent bio-mechanical performance [2]. It has been reported that the most popular class of Ti alloys, Ti6Al4V, suffers from poor tribological properties and is mainly used for the restricted non-tribological applications [3]. Further, in [4], it has been reported that the surface flaws can lead to the implant failure due to propagation of the cracks. The intrinsic characteristics of this alloy tend to release aluminum and vanadium ions, which results in their accumulations on the host tissues and causes toxic reactions [5,6]. Underlining such facts, many research interests are focused on the development of an effective alternative, β-phase Ti-Nb-Ta-Zr (β-TNTZ) biomedical alloy, for orthopedic implants, especially actabular cup, shoulder joint, and knee joint assemblies [7,8,9,10,11,12,13,14,15,16].

However, the poor finishability of the β-TNTZ alloy is one of critical barriers that limit the performance quality by attracting bacterial infection [17] and vulnerability to attract plaques [18], which entail inflammation around the implant surface [19,20,21]. Indeed, a wide range of finishing processes, including grinding [22], honing [23], ball burnishing [24], flexible abrasive tools [25], etc., have been developed for processing the free-form surfaces of the developed implants. As per [26], the manual finishing of the implant surfaces is non-effective, imprecise, and takes more time. Apart from the conventional finishing processes, the chemical mechanical polishing is effective in polishing Ti implants [27] and to obtain mirror-polished surfaces without any contamination and reacted layers [28]. Furthermore, polishing techniques such as electro-polishing, magneto-electro-polishing [29], and electron beam radiation [30] are useful to surface finishing in nano-scale.

The magneto-rheological fluid assisted abrasive finishing (MRF-AF) process has been successful in producing nano-finished precise components [26]. Further, the finishing of β-phase Ti-Nb-Ta-Zr alloy is difficult because of low surface hardness as compared to other biomaterial [31]. Therefore, in the present study, the in-house developed β-phase Ti-Nb-Ta-Zr alloy has been heat-treated to increase the surface hardness of the alloy and to make it suitable for abrasive finishing. The magneto-rheological (MR) fluid used as a polishing medium consisted of carbonyl-iron-particles (CIP) and hard-abrasive-powder particles in base medium of synthetic mineral oil and grease. The rheological characteristics and the yield strength of the developed MR fluid affects by the externally applied magnetic field [32]. Ultimately, the CIP in the MR fluid develops an interlinked chain along the direction of magnetic field, resulting in a semisolid abrasive tool to process hard surfaces [33]. Barman et al. studied the effect of magnetorheological polishing fluid compositions on the surface finish of Ti-alloy. Ultra-fine surface roughness, ranging 10–70 nm, has been achieved using different types of rheological fluids [34]. Barman et al. studied the effect of tool paths such as spiral and raster on the surface finishing of Ti-based bio-medical alloy. It has been observed that at tool rotational speed of 1200 rpm, working gap of 1 mm, and finishing time of 6.30 h, using a raster path provided the best surface finish and surface topography [35]. Parameswari et al. studied the effect of abrasive particle concentration on surface finishing. The finishing rate has been significantly affected by initial roughness and concentration of abrasive particles [36]. Nagdeve et al. developed a rotational-magnetorheological abrasive flow finishing (R-MRAFF) process based special tool for nano-finishing of femoral component of knee joint and surface finish in the range of 78–89 nm was attained, by considering the effect of various input process parameters [37]. Barman et al. studied the influence of magnetic field-assisted finishing (MFAF) process on the various surface finishing and the average surface roughness obtained was 11.32 nm. The roughness parameters have obtained the values in the range of nano-meters and rendered better surface topography [38].

From the available literature, the nano-finishing of β-phase Ti-Nb-Ta-Zr biomedical alloy has not been reported yet. The novelty of research work is that the high-strength β-phase Ti-Nb-Ta-Zr is very tough to process using convectional finishing processes. The MR-fluid based abrasive-finishing set was developed in-house and the capability of nano-finishing on heat-treated β-TNTZ substrate has been investigated using single and multi-objective optimization. The material removal (MR) and percentage change in surface roughness (%ΔRa) of the implant surface have been studied in response to input process parameters, such as carbonyl iron particles (CIP) concentration, rotational speed (Nt), and working gap (Gp). Further, the surface morphology and rendered image analysis have been performed to obtain the characteristics of the processed surfaces. The simulated body fluid (SBF) test has also been carried out to identify the corrosion resistivity of the MRF-AF finished β-TNTZ substrate specimens. Further, the as-corroded surfaces have been characterized to observe the effect of surface roughness on the achieved corrosion characteristics.

## 2. Materials and Methods

**Figure 1:**
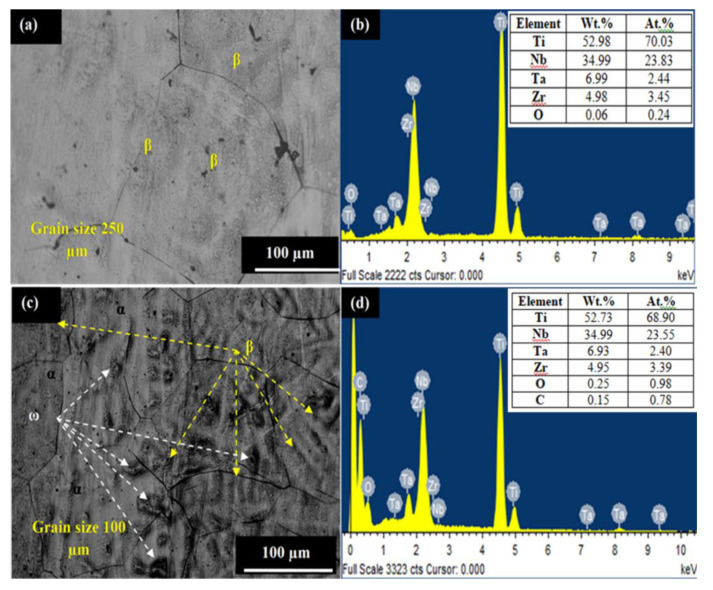
Microstructure and EDS spectrum: (**a**,**b**) β-TNTZ and (**c**,**d**) β-TNTZ after heat treatment.

High-strength β-phase Ti-Nb-Ta-Zr alloy has been developed using vacuum-arc melting process. The samples of size 10 × 5 mm for the finishing process were cut from the as-developed ingot through a wire-cut electric discharge machining process (Model Ecocut, Electronica, India). After that, the prepared specimens were subjected to a heat-treatment process to improve the mechanical properties of β-phase Ti-Nb-Ta-Zr as reported in previous study [39]. The microstructure of the samples before and after heat-treatment were examined by field emission scanning electron micrograph (FE-SEM; JEOL 7600F; JEOL Inc., Peabody, MA, USA) and associated energy dispersive spectroscopy (EDS, FE-SEM; JEOL 7600F; JEOL Inc., Peabody, MA, USA). From the microstructure analysis of untreated samples, it has been observed that the material comprised majorly β-type phases with grain size 250 µm, as can be seen in Figure 1a. The related EDS spectrum conform to the elemental composition and wt.% of each elements present in the material; refer to Figure 1b. After heat treatment, microstructure is refined and grain size becomes finer in the range of 100–150 µm; refer to Figure 1c. The heat-treated microstructure comprised α-type and ω-type phases, which further improved the mechanical properties of alloy. As a result, the ultimate compressive-strength and surface-hardness of the developed alloy was enhanced to 1195 MPa and 515 HV, respectively, as suitable for load-bearing implants requisites. Figure 1d shows the EDS spectrum and elemental composition of alloy after heat-treatment. The observations are close with the previous research studies [39,40,41,42].

**Figure 2:**
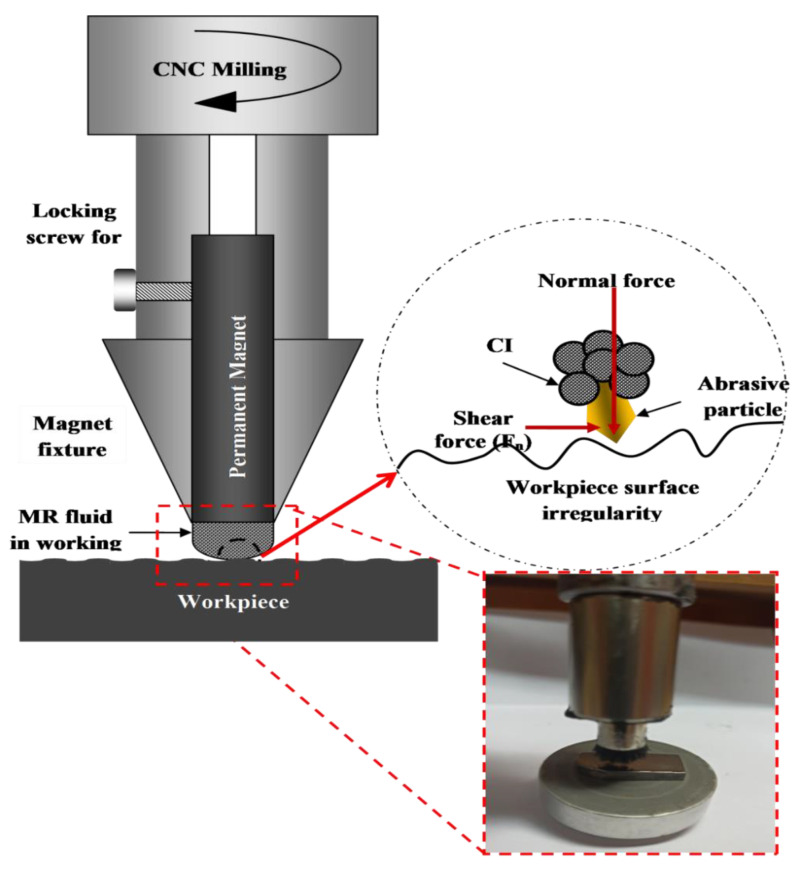
Pictorial representation of MRF-AF setup.

The heat-treated β-phase Ti-Nb-Ta-Zr alloy specimens were then finished using an in-house developed MRF-AF setup; refer to Figure 2. The MRF-AF processing consisted of three stages, such as development of magnetorheological-fluid, preparation of customized finishing magnetic assisted tool, and finishing of the work surfaces. A permanent magnet tool of material neodymium-iron (Nd-Fe-B) with magnetic flux intensity ~0.45 Taxella Gauss was used as tool for experimentation to provide the required magnetic field in the finishing zone. Generally, the working gap between the abrasive tool and β-phase Ti-Nb-Ta-Zr alloy workpiece was filled with the abrasive media that acted as a ball-end polishing brush. The speed at which the tool rotates plays a crucial role in attaining the required cutting forces to chip out the small amount of material from the work surface. Table 1 shows the process parameters and their levels.

Presently, the three most crucial input process parameters of MRF-AF process have been selected (such as CIP, Nt, and Gp) to identify their impact on achieved MR and Ra. The MR from the work surface has been calculated by using Equation (1):

(1) $$MR=\;\rho_{workpice}\times V_{total\;material\;removed}$$

where, $\rho_{workpice}$ is the density of the workpiece and $V_{total\;material\;removed}$ is the total volume of material removed. Digital weighing balance (Scientech, Delhi, India) of accuracy 0.01 mg was used for the MR calculations. Further, the Ra values of the as-finished work surfaces have been calculated by using a non-contact three-dimensional (3D) Surface Profilometer (Talysurf CCI Lite, Leicester, UK) that uses a white light interferometer equipped with the TalyMap Platinum 6.0. The measurement of Ra was taken at three different locations. The design of experimentation technique, based on Taguchi L9 orthogonal array, was used to perform the statistical analysis on the observed output responses (such as MR and Ra), and to identify the statistical importance of the selected input process parametric levels on the observed responses using analysis of variance (ANOVA) [43]. Table 2 illustrates the control log of experimentation. Furthermore, corrosion performance parameter, corrosion-current, of the as-finished β-phase Ti-Nb-Ta-Zr alloy specimens has been studied in SBF medium using potentiodynamic polarization-based electrochemical system-1000E (make: Gamry Instruments, Warminster, PA, USA). The concentration of the SBF medium (pH 7.2) consisted of 9, 0.24, 0.43, and 0.2 g/L of NaCl, CaCl_2_, KCl, and NaHCO_3_ [44]. For this, potential rate and scan range has been selected as 1 mV/s and −250OCP to +250OCP mV, respectively. Tafel extrapolation technique was used to calculate the corrosion-current (ICOR). Before evaluating ICOR, the specimens were dipped in the SBF solution for about 24 h and the test was conducted at 37 ± 0.1 °C.

## 3. Results and Discussion

### 3.1. Parametric Optimization

In the present research work, to understand the effect of the input process parameters on the material removal (MR) and surface roughness (Ra), has been studied through the use of design of experimentation. The concentration of the diamond abrasive particles has been selected as 3.5%vol. on the basis of pilot experimentation. As noticed, the 3.5%vol. of the diamond abrasive particles corresponded to the higher MR and lower Ra. The output responses observed after performing the set of experimentations, following Table 3, have been given in Table 3. The signal/noise (S/N) ratio has been calculated using Minitab-17 statistical software package. In the case of MR, the S/N ratio has been optimized at “larger-the-better” options, whereas in the case of Ra, the S/N ratio has been optimized at “smaller-the-better” option.

**Figure 3:**
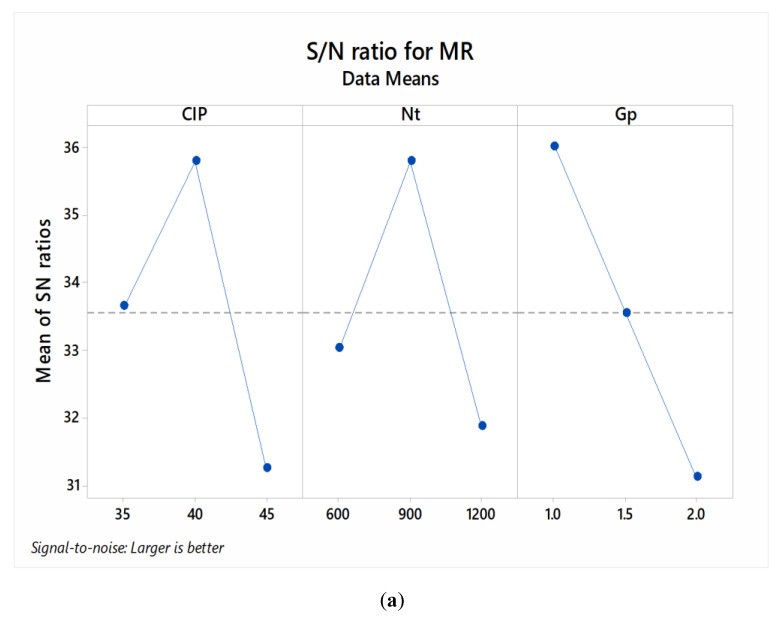

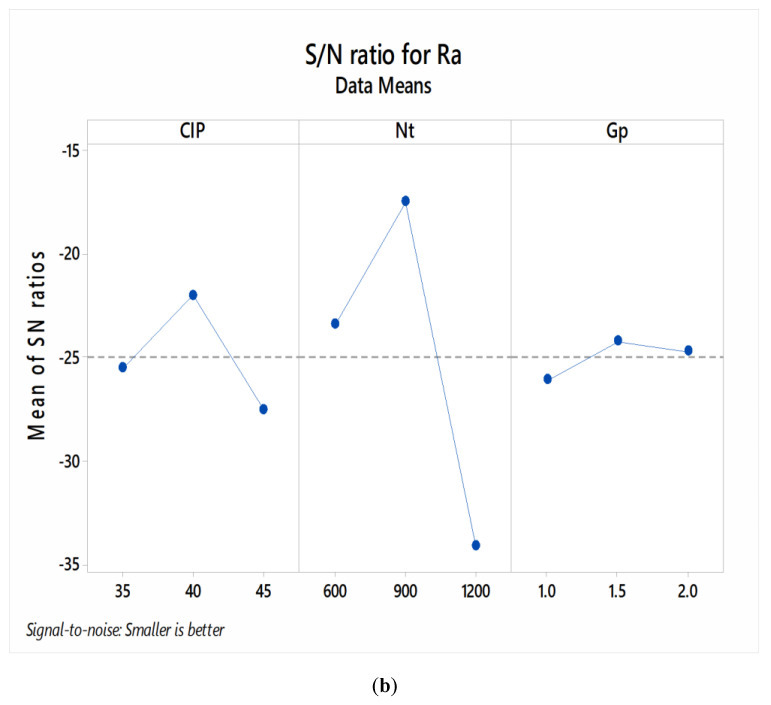
S/N ratio response of the input process parameters on MR (**a**) and Ra (**b**).

Figure 3 shows the S/N ratio plot for the MR of the abrasive finished β-phase Ti-Nb-Ta-Zr alloy. It can be seen in the case of process parameter ‘CIP’ that the MR increased by increasing the concentration of the iron particles in the MR fluid from 35 to 40%vol. This is mainly due to the fact that, as the concentration of iron particles increased, the abrasive tool became stronger and more efficient to process the hard work surfaces. The iron particles are the prime source of producing a magnetically held semi-solid tool; hence, the CIP proportion of 40%vol. was favoured in obtaining stronger abrasive tools capable of withstanding higher cutting forces executed while processing the surface. However, as the CIP proportion was further increased to 45%vol., the MR has reduced drastically. This is mainly due to the fact that the higher proportion of iron particles has dominated the existence of abrasive diamond particles, as a result of the abrasive particles trapped within the rich iron particles. Owing to this, the cutting action of the resulting abrasive tool has been sacrificed. Further, in the case of “Nt”, it can be seen that the MR has first increased by increasing the rotational speed from 600 to 900 rpm; however, with a further increase in the rotational speed to 1200 rpm, the MR has been dropped, significantly. Noticeably, at 900 rpm, the abrasive tool exerted greater cutting forces on the work material and therefore resulted in greater material removal. Further increase in the rotational speed to 1200 rpm has widened the magnetic flux density area and impeded the strength of the abrasive cutting tool. As regards to “Gp”, it has been seen that when the working gap has been increased from 1 to 2 mm, the MR of the MRF-AF processed β-phase Ti-Nb-Ta-Zr alloy reduced. This can be attributed towards the reason that due to an increase in the working gap, the normal tangential force exerted by the abrasive cutting tool on work surface has reduced, resulting in weak cutting action.

**Figure 4:**
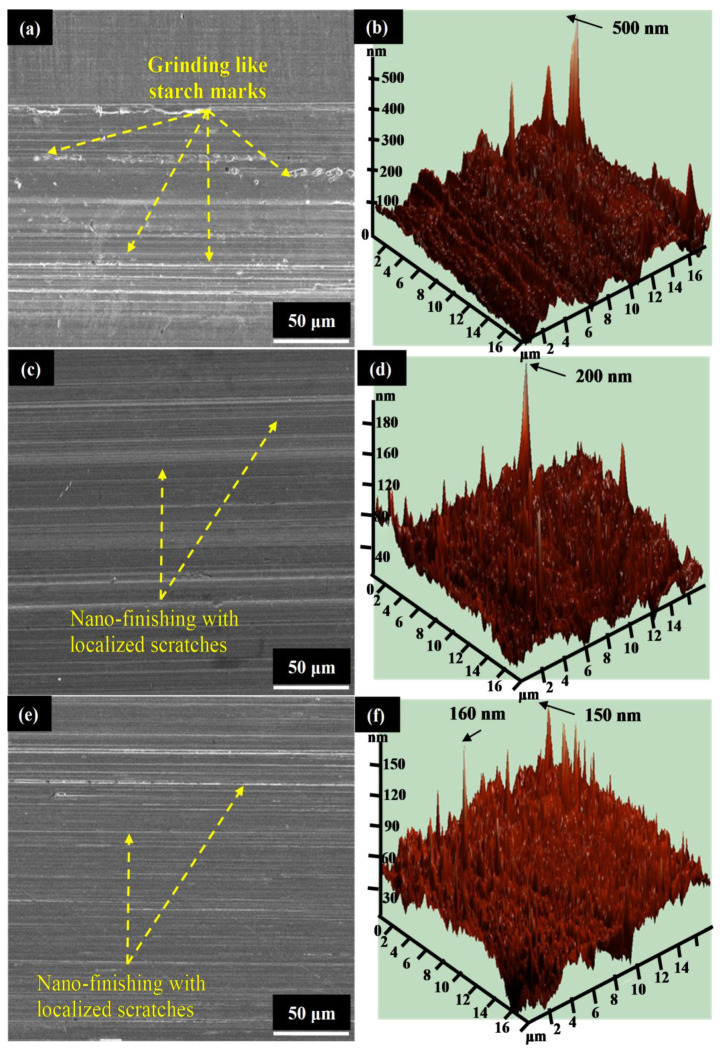
Surface morphology and AFM imaging of MRF-AF processed alloy surface (**a**,**b**) at 600 rpm, (**c**,**d**) at 1200 rpm, and (**e**,**f**) at 900 rpm.

Further, in the case of ‘Ra’, it has been found that the surface roughness of the MRF-AF processed β-phase Ti-Nb-Ta-Zr alloy reduced by increasing the CIP process parametric level from 35 to 40%vol. This is due to the fact that, with an increase in the CIP level to 40%vol., the magnetic flux density of the abrasive tool has increased, resulting in an increase in the cutting strength of the abrasive finishing tool. However, by further increasing the CIP to 45%vol., the cutting action of the diamond abrasive particles has been dropped due to undesirable increase in the content of iron particles in the abrasive tool. Further, in the case of “Nt”, it can be seen that the surface finishing of the processed alloy increases by increasing the rotational speed from 600 to 900 rpm. The reason behind this observation is the same as discussed for the MR. At 900 rpm, the cutting thrust force exerted by the developed abrasive tool has increased to result in the removal of the peak from the surface of the alloy. With the increase in the rotational speed the cutting action of the abrasive particles reduced [45,46]. At 1200 rpm, the brush of abrasive particles flared due to that magnetic flux density area increased as a result the materials removal reduced. The surface morphology and atomic force microscopy (AFM) analysis of the MRF-AF processed β-phase alloy—refer to Figure 4—also indicate that the presence of rough surface textures on the metallic implant surface are high. There are severe surface scratches on the metallic surface at 600 and 1200 rpm. However, comparatively, the surface is quite finished in the case of 900 rpm. Furthermore, it can be seen that, in the case of 900 rpm, the maximum heights of the peaks on the processed surfaces ranged from 150–160 nm. As regards the “Gp”, it can be seen that the surface finishing of the MRF-AF processed β-phase alloy increased by increasing the work gap from 1 to 1.5 mm and further underwent a slight drop after increasing the work gap to 2.0 mm. This is due to the fact that, when the processing work gap has been increased, the tangential cutting force on the work surface reduced that contained the cutting action of the abrasive tool only limited to the removal of the surface peaks.

Table 4 shows the analysis of variance (ANOVA) analysis of S/N ration for MR and Ra. It can be seen that, in the case of MR, all the input process parameters are significant at 95% confidence level as their respective *p*-value is less than 5%. Further, the percentage contribution of input process parameters, such as CIP, Nt, and Gp is 33.64, 26.44, 39.28%, respectively. In the case of Ra, it has been found that only Nt (*p* < 0.05) has obtained statistically significant effect on the surface roughness of the processed metallic alloy. Further, process parameters such as CIP, Nt, and Gp has obtained 9.39, 85.57, and 1.12% contribution for Ra. The optimized levels of the input process parameters in response of MR are: CIP—45%vol., Nt—900 rpm, and Gp—1 mm. Whereas, in the case of Ra, the optimized input process parametric levels are: CIP—45%vol., Nt—900 rpm, and Gp—1.5 mm. Further, Table 5 shows the response table of S/N ratio for various levels of selected input process parameters. These given values have been used for the prediction of optimized response of the output parameters as per the suggested optimum levels of the input processing parameters.

In order to confirm the accuracy of the predicted results, a set of five confirmatory experiments have been conducted on the suggested parametric levels of input parameters. Table 6 shows the average values of responses on confirmatory experimental results.

It can be seen that the confirmatory experimentation results are in good agreement with the predicted results, highlighting the accuracy of the applied design of experimentation approach in obtaining the desirable output responses.

### 3.2. Corrosion Performance

Reportedly, the resulting surface topographical features of biomedical implant plays a significant role in the osteoblast adhesion, differentiation, extracellular matrix secret, and corrosion resistance [47,48,49]. Further, the nanostructured implant surface, being conductive to the body fluid, accelerates the osseointegration. There are numerous reports indicating that the synergistic effects of microstructure and nanostructure are often desirable to mediate the mechanism of cell attachment, growth, differentiation, and appetite formation [50]. Underlining these facts, the present study investigates the effect of the MRF-AF processing of β-phase Ti-Nb-Ta-Zr alloy to obtain nano-scale surface finish to enhance the biological performance of the resulting implant surface, especially to identify the obvious differences of corrosion resistance in various obtained surface. As the corrosion performance of the finished implants mainly depends on the quality of surfaces (Ra value) produced after MRF-AF processing, the statistical analysis (as presented in Table 5) has been used for performing the SBF test runs. Three different categories of test runs have been conducted to visualize the impact of selected input processing parameters (CIP, Nt, and Gp) and on ICOR.

**Figure 5:**
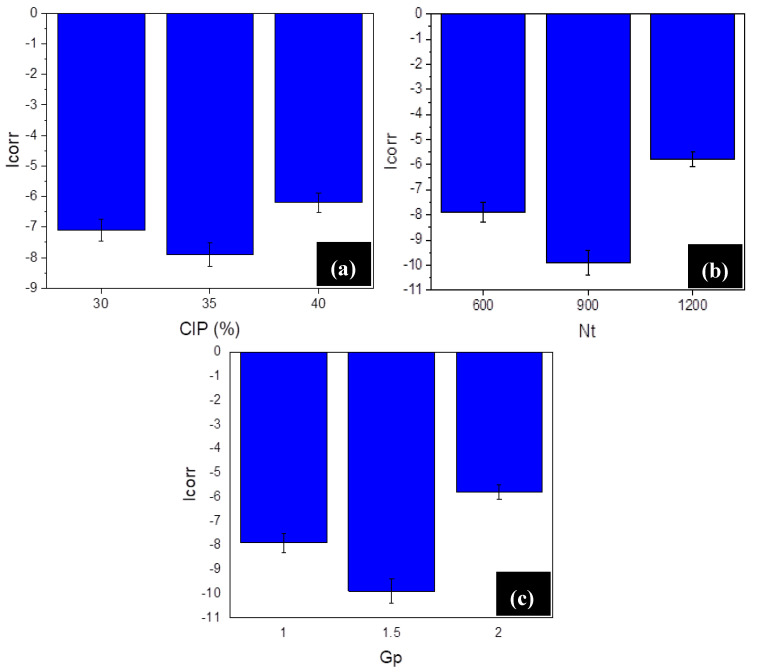
Results of ICOR for set #1 (**a**), set #2 (**b**), and set #3 (**c**). (Note: The unit of ICOR is μA/cm^2^).

The set #1 consisted of three samples with variable parameter ‘CIP’ at three selected levels (35, 40, and 45%vol.) and considering the optimum level the others (Nt, and Gp) as per Figure 3b. Further, the set #2 contains the three samples at three different levels of Nt (600, 900, and 1200 rpm) and considering the optimum levels of CIP and Gp. Lastly, the set #3 contains the three samples corresponding to three levels of Gp (1.0, 1.5, and 2.0 mm), while taking the levels of CIP and Nt as optimum. The corresponding plot for the ICOR is presented in Figure 5. From Figure 5, it can be seen that the highest corrosion resistance in the case of set #1 (ICOR~−8 μA/cm^2^) has been corresponded to CIP of 40%vol. The observed trend in-line with the observation made while the optimization of Ra response parameter. Further in the case of set #2, it has been found the parametric levels of Nt has maximum impact on the obtained values of the ICOR. As observed, at 900 rpm minimum corrosion-current value has been obtained, signifying it as the best parametric level for obtaining the highest possible corrosion resistance. This particular parameter has been previously identified as statistically significant; therefore, to elaborate further the significance of Nt, a morphological study on the set #3 samples has been performed.

**Figure 6:**
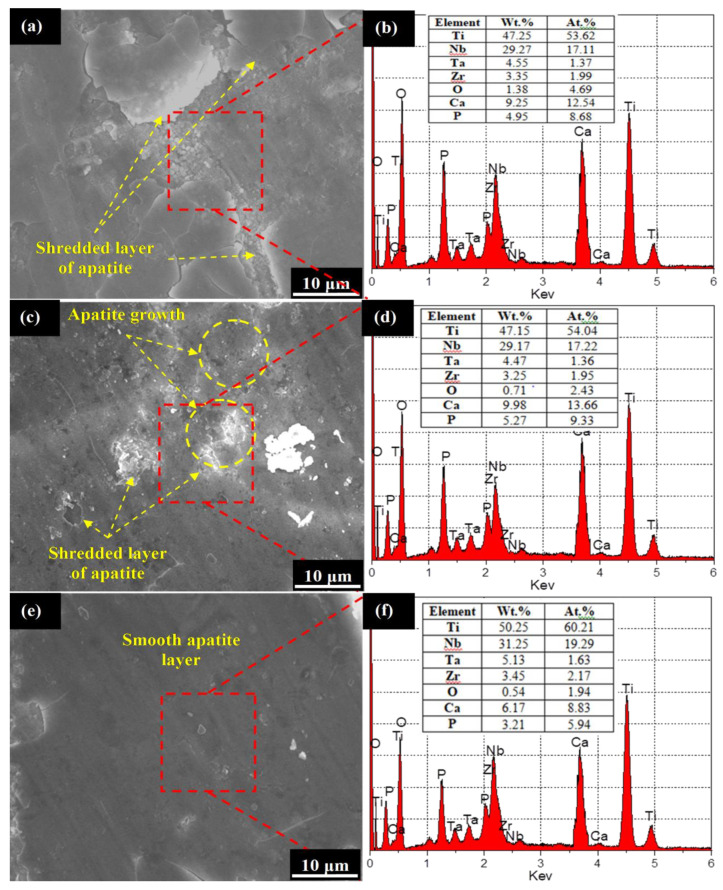
Corroded morphology and EDS spectrum of finished surface: (**a**,**b**) 600 rpm, (**c**,**d**) 1200 rpm, and (**e**,**f**) 900 rpm.

Figure 6 shows the corroded surface morphology and associated EDS spectrum of the set #2 samples. It can be seen from the Figure 6a that the surface has undergone severe shredding of the obtained apatite layer owing to the rough nano-texture. The rough sites existing on the surface have acted as nuclei sites to originate the corrosion mechanism. The corresponding higher ICOR values have subsisted the applied potential resulting in pitting and galvanic corrosion. The corresponding EDS spectrum (refer to Figure 6b) indicated the formation of ‘O’, ‘P’, and ‘Ca’ elements representing the existence of non-uniformly formed apatite layer. Further, in the case of 1200 rpm, it can be seen from Figure 6c that, although apatite layer has formed on the finished implant surface, due to higher ICOR value the brittle fracture and erosion of the apatite layer has been witnessed. The apatite layer acts as a barrier to the corrosion but severe shredding and higher ICOR value forced the formed apatite layer to undergo brittle fracture and its removal from the implant’s surface. Indeed, such phenomenon is never desirable for biomedical implant due to the possibilities of biological complications (such as toxicity and hypersensitivity) owing to the release of metal ions. However, as regards 900 rpm, no shredding, pit formation, and galvanic corrosion has been identified in the case of Figure 6e. This is primarily due to the fact that, at 900 rpm, a very fine finished surface has been obtained that encouraged the formation of uniform apatite layer to act as a corrosion barrier. Further the observed ICOR value in this case is minimum (~ −10 μA/cm^2^) that diminished the formation of electric potential.

## 4. Conclusions

In the present study, an investigation has been made to analyze the influence of the input process parameters of MRF-AF process on the MR and Ra of the finished β-phase Ti-Nb-Ta-Zr alloy. Besides this, the influence of the input process parameters on corrosion-resistance of the finished samples has also been studied. Based on the key findings, the following conclusions can be drawn:

It has been found that the MR of the processed alloy specimens has been significantly affected by all the selected input process parameters of MRF-AF. Furthermore, the optimized parametric levels as regards to MR are: CIP—40%vol., Nt—900 rpm, and Gp—1.0 mm.

However, in the case of Ra, it has been found that except Nt, none of the input process parameters are statistically significant. In this case, the optimized parametric levels identified are: CIP—40%vol., Nt—900 rpm, and Gp—1.5 mm. The confirmatory experimentation results have been found in good correlation with the predicted responses. 

The results of the corrosion analysis of the developed samples highlighted that the corrosion resistance of the finished samples depends on their surface topography. It has been found that the samples possessed high surface finish developed a uniform layer of apatite in SBF medium that performed as a corrosion barrier. On the other side, the rough sites on the implant surface acted as the nuclei to propagate the corrosion mechanics that later resulted in shredding, pitting, and galvanic corrosion.

Overall, the results highlight that the MRF-AF process is highly suitable for producing nano-scale finishing of the biomedical implants made of high-strength β-phase Ti-Nb-Ta-Zr alloy.

## Figures and Tables

**Table 1:** MRF-AF process parameters and their working levels.

Symbol	Process Parameters	Unit	Range
CIP	Iron (Fe); size~25 µm	% by vol.	30, 35, 40
Nt	Rotational speed of tool	rpm	600, 900, 1200
Gp	Work-gap	Mm	1, 1.5, 2

**Table 2:** Control log of experimentation.

Experiment No.	CIP	Nt	Gp
1	35	600	1.0
2	35	900	1.5
3	35	1200	2.0
4	40	600	1.5
5	40	900	2.0
6	40	1200	1.0
7	45	600	2.0
8	45	900	1.0
9	45	1200	1.5

**Table 3:** Control log of experimentation.

Experiment No.	MR					Ra				
	Raw Value (g)			Mean	S/N	Raw Value (nm)			Mean	S/N
**1**	60	50	70	60	35.56	16	15	11	14	−22.92
**2**	50	55	75	60	35.56	8	10	6	8	−18.06
**3**	28	35	30	31	29.83	60	55	65	60	−35.56
**4**	68	55	57	60	35.56	10	12	8	10	−20.00
**5**	65	50	65	60	35.56	4	2	6	4	−12.04
**6**	68	67	60	65	36.26	45	50	40	45	−33.06
**7**	23	27	25	25	27.96	21	20	22	21	−26.44
**8**	66	62	67	65	36.26	14	15	10	13	−22.28
**9**	30	28	32	30	29.54	75	50	55	60	−35.56
Overall mean S/N ratio (mo)					33.56					25.10

Note: The unit of S/N ratio is decibel (dB).

**Table 4:** ANOVA for S/N ratio for MR and Ra.

Source	Degree of Freedom	Sum of Square	Variance	F-Test	*p*-Value	Contribution (%)
MR						
CIP	2	30.9716	15.4858	53.19	0.018 *	33.64
Nt	2	24.3382	12.1691	41.80	0.023 *	26.44
Gp	2	36.1651	18.0825	62.11	0.016 *	39.28
Residual Error	2	0.5823	0.2912			0.632
Total	8	92.0573				100
Ra						
CIP	2	47.086	23.543	2.40	0.294	9.39
Nt	2	429.047	214.523	21.85	0.044 *	85.57
Gp	2	5.630	2.815	0.29	0.777	1.12
Residual Error	2	19.639	9.819			3.91
Total	8	501.401				100

* Indicates significant parameters, F-test is the Fisher’s test, and *p*-value is the probability.

**Table 5:** S/N response of input process parameters.

Level	CIP	Nt	Gp
MR			
1	33.65	33.03	36.03 *
2	35.79 *	35.79 *	33.56
3	31.25	31.88	31.12
Delta	4.54	3.92	4.91
Rank	2	3	1
Ra			
1	−25.52	−23.40	−26.09
2	−21.98 *	−17.46 *	−24.23 *
3	−27.51	−34.14	−24.68
Delta	5.53	16.68	1.86
Rank	2	1	3

* Indicates the optimum parameters.

**Table 6:** Predicted and confirmatory experimentation results at optimized levels of input parameters.

Responses	Predicted Values	Confirmatory Values	Difference (±)
MR (g)	105.8	103.7	2.1
Ra (nm)	4.63	4.67	0.04
